# A VIEW OF OLDER ADULTS’ DEMOGRAPHIC, HEALTH, AND ECONOMIC PROFILES BY RETIREMENT CHOICE STATUS

**DOI:** 10.1093/geroni/igz038.463

**Published:** 2019-11-08

**Authors:** Jane L Tavares, Marc A Cohen

**Affiliations:** 1 University of Massachusetts Boston Gerontology Institute, LeadingAge LTSS Center, Boston, Massachusetts, United States; 2 University of Massachusetts Boston Gerontology Institute, LeadingAge LTSS Center @UMass Boston, Boston, Massachusetts, United States

## Abstract

Gerontologist, economists, and other scientists have documented that involuntary or forced retirement is a more common occurrence among middle-aged adults than previously thought. Prevailing research has further indicated that involuntary retirement and off-time transitions are associated with poorer physical and mental well-being in later life. However, few studies have sought to gain a comprehensive understanding of who compromises the population of involuntary retirees and how these individuals compare to voluntary retirees. This study used the 2014 wave of the Health and Retirement Study (N=9,409) to analyze a United States representative sample of fully retired individuals aged 55 and older in order to develop a demographic, health, and economic profile of voluntary versus involuntary retirees. Analyses showed that that male, younger age group, minority race, separated/divorced, less educated, and south and west region residing individuals have significantly greater representation in the involuntary retirement group compared to the voluntary retirement group. Further, involuntary retirees reported significantly poorer physical and mental health and fewer financial resources than voluntary retirees. A supplementary longitudinal analyses also revealed that while health steadily declines for both of the retirement choice status groups, involuntary retirees maintained significantly poorer health than voluntary retirees over time. Observations from this study highlight the importance of identifying predictors of involuntary retirement and utilizing longitudinal analyses to gain greater insight into how health and economic characteristics factor into retirement decisions as well as how retirement decisions impact future health and economic characteristics.

